# Development of Nickel- and Magnetite-Promoted Carbonized Cellulose Bead-Supported Bimetallic Pd–Pt Catalysts for Hydrogenation of Chlorate Ions in Aqueous Solution

**DOI:** 10.3390/ijms222111846

**Published:** 2021-10-31

**Authors:** Emőke Sikora, Dániel Koncz-Horváth, Gábor Muránszky, Ferenc Kristály, Béla Fiser, Béla Viskolcz, László Vanyorek

**Affiliations:** 1 Institute of Chemistry, University of Miskolc, 3515 Miskolc-Egyetemváros, Hungary; kemsik@uni-miskolc.hu (E.S.); kemmug@uni-miskolc.hu (G.M.); kemfiser@uni-miskolc.hu (B.F.); bela.viskolcz@uni-miskolc.hu (B.V.); 2 Higher Education Industry Cooperation Centre, University of Miskolc, 3515 Miskolc-Egyetemváros, Hungary; femkhd@uni-miskolc.hu; 3 Institute of Mineralogy and Geology, University of Miskolc, 3515 Miskolc-Egyetemváros, Hungary; askkf@uni-miskolc.hu; 4 Ferenc Rákóczi II, Transcarpathian Hungarian College of Higher Education, 90200 Beregszász, Ukraine

**Keywords:** Pd–Pt clusters, chlorate reduction, magnetite

## Abstract

Cellulose grains were carbonized and applied as catalyst supports for nickel- and magnetite-promoted bimetallic palladium- and platinum-containing catalysts. The bimetallic spherical aggregates of Pd and Pt particles were created to enhance the synergistic effect among the precious metals during catalytic processes. As a first step, the cellulose bead-based supports were impregnated by nitrate salts of nickel and iron and carbonized at 973 K. After this step, the nickel was in an elemental state, while the iron was in a magnetite form in the corresponding supports. Then, Pd and Pt particles were deposited onto the supports and the catalyst surface; precious metal nanoparticles (10–20 nm) were clustered inside spherical aggregated particles 500–600 nm in size. The final bimetallic catalysts (i.e., Pd–Pt/CCB, Pd–Pt/Ni–CCB, and Pd–Pt/Fe_3_O_4_–CCB) were tested in hydrogenation of chlorate ions in the aqueous phase. For the nickel-promoted Pd–Pt catalyst, a >99% chlorate conversion was reached after 45 min at 80 °C. In contrast, the magnetite-promoted sample reached an 84.6% chlorate conversion after 3 h. Reuse tests were also carried out with the catalysts, and in the case of Pd–Pt/Ni–CCB after five cycles, the catalytic activity only decreased by ~7% which proves the stability of the system.

## 1. Introduction

The elimination of chlorate ($ClO_{3}^{-}$) from industrial process water is necessary not only from an environmental but also from an industrial point of view [1,2,3,4]. In the field of chlor-alkali electrolysis, the first membranes were introduced in the 1970s, which provided a more efficient ion-selective space-separation than the previously used diaphragm [5]. The two main disadvantages of the process that includes a membrane cell are the formation of chlorate and the sensitivity of the applied membranes to impurities [6]. During chlor-alkali electrolysis, the chlorate-contaminated brine has a negative effect on the quality of caustic soda and could also damage the membranes and decrease the efficiency of the procedure [3]. One solution to reduce the chlorate concentration is catalytic hydrogenation [1,3,7]. The main advantage of this procedure—compared to the currently used thermal hydrochloric acid treatment—is that small amounts of additional chemicals are enough to achieve the desired effect [4,8].

However, membranes are extremely sensitive not only to chlorate but also to other elements, especially to metals such as Ca, Mg, and Al [9]. This should also be considered in the selection of the applied catalytic system in order to minimize the input of contaminants into the technology. It is also important to use a catalyst with a macroscopic structure, since the separation of finely divided powder from materials for the medium is difficult [10,11]. Therefore, easily separable support materials must be used to avoid this issue.

Carbon materials are extremely popular supports not only in catalysis but also in many other fields due to the fact of their electrical conductivity, chemical resistance, relatively high specific surface area, and, in some cases, good separation [12,13]. As a renewable resource, cellulose is preferred as a starting material to prepare functional carbon materials [14]. Therefore cellulose and carbonized cellulose (CC) have already been successfully tested in various applications, where they have been used as a microwave absorber [15], flexible electrode [16], anode for high-performance lithium ion batteries [17], and catalyst support [18]. Carbon-supported platinum group metal (Pd, Pt, Ru, Ir, and Rh) catalysts showed outstanding results in chlorate reduction [19,20,21]. Several studies have confirmed that catalysts made by combining these precious metals, such as bimetallic Pd–Pt systems, generally perform better than their monometallic counterparts due to the synergistic effects [22,23,24,25].

By studying hydrodesulfurization reactions, it was found that hydrogenation was faster with Pd-containing catalysts compared to their Pt-containing counterparts due to the higher hydrogenation ability of palladium, but Pt catalyst had a higher desulfurization activity [26]. Furthermore, bimetallic catalyst was also synthesized and tested, and it was more active than expected from a simple combination of the monometallic Pd and Pt systems because of the chemical synergism between the noble metals [26]. Guerrero et al. came to a similar conclusion when they found that the activity of their Pd–Pt catalyst was twice as active as the corresponding monometallic Pt catalyst on an Al2O3–TiO2 support [27]. This synergistic effect was studied in the case of a USY zeolite-supported Pd–Pt catalyst, and a new bond was found that was completely different from the Pt–Pt and Pt–Pd metallic bonds. It has been established that in this catalyst, where the ratio of Pd:Pt is 4:1, this new bond reflects the ionic properties of Pt through the Pt–Pd interaction, and the bond has a cationic and sulfur tolerance property [28]. The synergistic effect was exploited by Sangmoon Byun et al. when they synthesized Pd–Pt–Fe3O4 nanoparticles [29]. This combined and magnetic catalyst has proven to be extremely durable (300 reuse tests) and has shown effective results in the reduction of nitro compounds to anilines or alkylamines. This durability could also be attributed to the combination of the two metals, as some studies have confirmed that supported Pd–Pt catalysts are more resistant to catalyst poisons such as sulfur [28,30].

In this study, cellulose grains were carbonized and applied as catalyst support for nickel- and magnetite-promoted palladium–platinum catalysts. The developed bimetallic catalysts were tested in chlorate hydrogenation in the aqueous phase to prove their applicability. 

## 2. Results and Discussion

### 2.1. Characterization of the Catalyst Supports

Thermogravimetric analysis was carried out on the impregnated and non-impregnated samples to monitor the cellulose carbonization process (Supplementary Materials Figure S1). The first weight loss occurred at approximately 375 K due to the evaporation of water. The subsequent large weight losses in the case of the iron nitrate, nickel nitrate-containing, and non-impregnated samples were 25.9 wt%, 22.3 wt%, and 19.2 wt%, respectively, which can be associated with the oxidation of carbon.

SEM images of the various CCBs shows that the surfaces of the Ni–CCBs were more homogenously covered with metal nanoparticles compared to the magnetite-containing beads (Supplementary Materials Figure S2A,D). In this sense, the dispersibility of nickel particles on the support was more homogenous.

**Figure 1:**
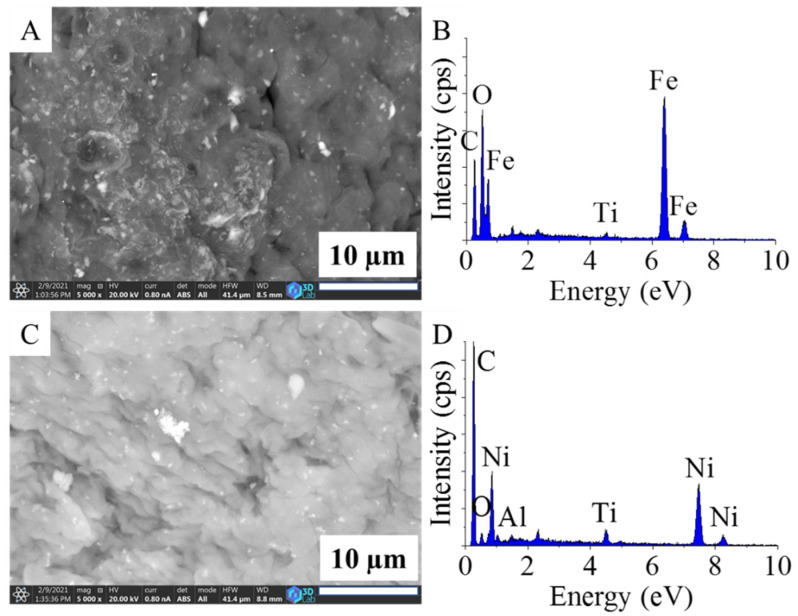
SEM images and EDS spectra of the Fe_3_O_4_–CCB (**A**,**B**) and Ni–CCB (**C**,**D**) catalyst supports.

The surface of the carbonized, magnetite-containing beads was richly coated with magnetite nanoparticles (Figure 1A). The crystallites were aggregated, and the particle sizes were large (7–10 µm), but smaller structures (1–2 µm and even 10–40 nm) were also visible on the surface (Supplementary Materials Figure S2). The size distribution in this sense was very inhomogeneous for the Fe_3_O_4_–CCB sample.

For the nickel-containing support, the size distribution of the Ni particles on the surface of the CCBs covered a wide range, from a few hundred nanometers to 10–20 microns (Figure 1C and Supplementary Materials Figure S2E,F).

The non-impregnated CCBs were also analyzed by SEM (Supplementary Materials Figure S3). Interestingly, fluffy, coral-like surfaces with small fibrous formations were found, which formed during the decomposition of cellulose. However, this was not experienced in the case of the impregnated samples, and it is assumed that the presence of metal nitrates led to the oxidation of these formations.

The EDS spectrum of Fe_3_O_4_–CCB verified the presence of oxygen and iron which originated from the magnetite (Figure 1B). Titanium was also detected in the carbon matrix, which indicates the presence of TiO_2_ and can be explained by the synthesis protocol of the Mavicell cellulose beads [31].

The presence of nickel in Ni–CCB was confirmed (Figure 1D). Titanium was detected in this case as well and, in addition, Al was also found, which can be associated with the sample holder of the SEM. There was also a smaller but visible peak of oxygen, which indicates that oxygen-containing functional groups were also present in the sample. To identify these functional groups, FTIR measurements were conducted.

**Figure 2:**
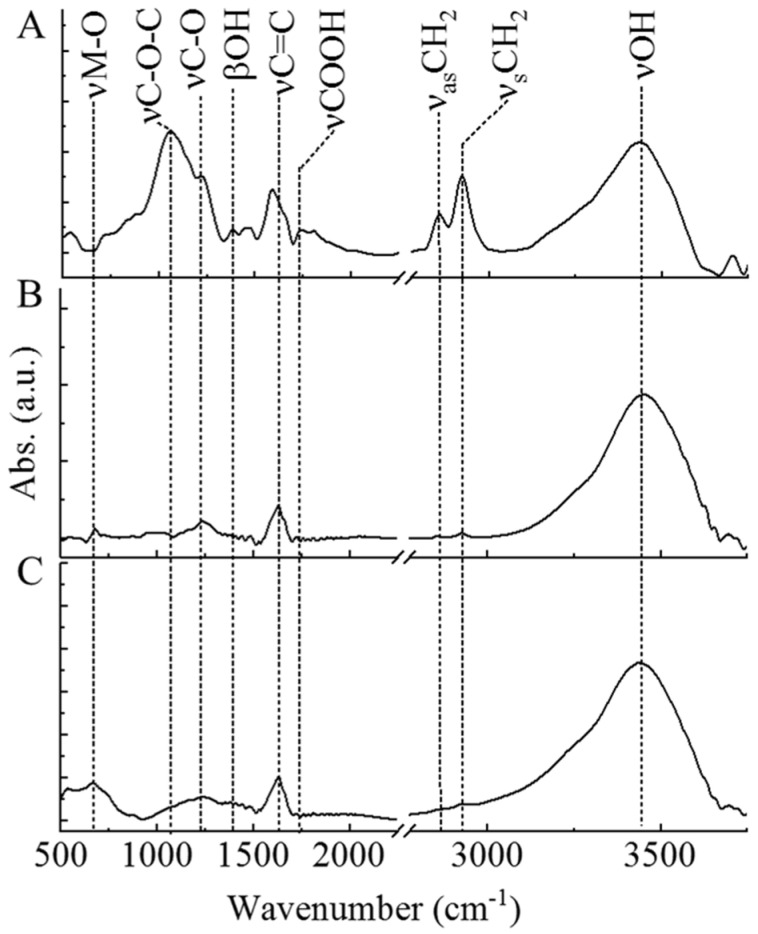
FTIR spectra of the non-impregnated (**A**), Ni- (**B**), and Fe_3_O_4_-containing (**C**) carbonized cellulose beads (CCBs).

On the FTIR spectrum of the non-impregnated CCB sample, several oxygen containing functional groups were identified (Figure 2A). At the 3454 cm^−1^ wavenumber, a peak was located that was associated with the stretching vibration mode of the hydroxyl groups. The bending mode of hydroxyl groups (βOH) was also identified at 1392 cm^−1^.

The presence of other oxygen-containing functional groups was verified, as two more peaks at 1228 cm^−1^ and 1058 cm^−1^ were located on the infrared spectrum of the carbon beads that corresponded to the stretching vibration mode of the C–O–C and the C–O bonds. Furthermore, the presence of surface carboxyl functional groups (νCOOH) was also proved (Figure 2A, 1734 cm^−1^). The band at 1590 cm^−1^ can be linked to νC = C which represents the skeletal vibration of the carbon matrix. The symmetric (2924 cm^−1^) and asymmetric (2855 cm^−1^) vibration modes of the CH_2_ groups were also located in two well separated bands. The identified oxygen-containing functional groups contributed to the hydrophilic character of the carbonized cellulose beads and improved their wettability in the aqueous phase. The carboxyl and hydroxyl groups can be deprotonated in the aqueous phase which will lead to negative electrokinetic potential. Thus, the negatively charged surface of the beads and the metal cations can establish electrostatic interactions. In this sense, these groups are beneficial and capable of anchoring the metal ions and, thus, play a role in the catalyst preparation processes. After carbonization, the intensity of the peaks on the spectra of the nickel(II) nitrate- and iron(III) nitrate-impregnated cellulose beads was smaller compared to the non-impregnated CCBs. A metal–oxygen vibration mode was also identified between the 500 and 700 cm^−1^ wavenumbers. In the Fe_3_O_4_–CCB sample, the wide νOH band also included a hydroxyl bond of magnetite and adsorbed water. The band of the νC=C vibration showed a shift in the case of the nickel- and magnetite-containing carbon samples, which suggests the formation of π backdonation (π–d) interaction between the metal (and metal oxide) particles and carbon beads.

**Figure 3:**
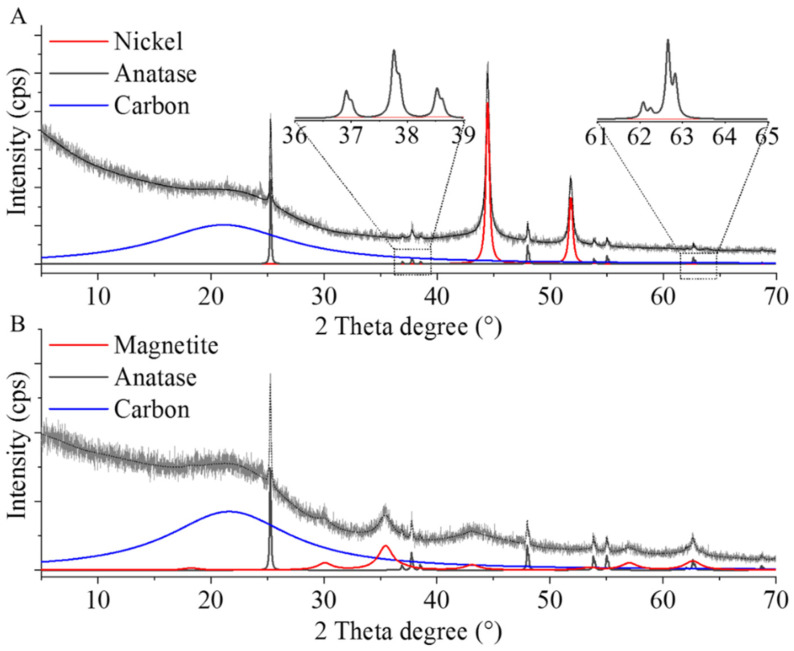
XRD patterns of the Ni- (**A**) and magnetite (Fe_3_O_4_)-containing (**B**) carbonized cellulose supports.

The carbon supports were also examined by XRD (Figure 3). On the diffractogram of the nickel-containing carbon beads, peaks were identified at 44.5° and 51.8°, two theta degrees, which were associated with the Ni(111) and Ni(200) reflexions of elemental nickel (JCPDS Card No. 04-0850). Anatase reflexions were also identified at 25.3° (101), 36.9° (103), 37.8° (004), 38.5° (112), 48.0° (200), 53.9° (105), 55.0° (211), and 62.7° (204), two theta degrees (JCPDS Card No. 21-1272). The presence of anatase can be explained by the production of the Mavicell beads. The 002 reflexion of carbon is shown at 21.1°, two theta degrees (JCPSD Card. No. 41-1487).

The XRD pattern of the Fe_3_O_4_–CCB sample confirmed that the iron was in the magnetite (Fe_3_O_4_) form (Figure 3B). Peaks at 18.3°, 30.1°, 35.4°, 43.1°, 57.1°, and 62.7°, two theta degrees, were associated with the (111), (220), (311) (400), (511), and (440) reflexions of synthetic magnetite. Reflexions corresponding to anatase and the wide peak (002) of carbon were identified on the diffractogram of this sample as well.

### 2.2. Characterization of the Pd–Pt/CCB, Pd–Pt/Ni–CCB, and Pd–Pt/Fe_3_O_4_–CCB Catalysts

The surface area of the prepared catalysts were determined, and it was found that the Pd–Pt/Ni–CCB sample had the highest (431 m^2^∙g^−1^), while the Pd–Pt/CCB sample had the lowest (379 m^2^∙g^−1^). The Pd–Pt/Fe_3_O_4_–CCB catalyst was right in between the other two samples in terms of surface area (408 m^2^∙g^−1^). The metal content of the final catalysts was measured using ICP-OES (Table 1).

Based on the results, the Ni–CCB and Fe_3_O_4_–CCB were able to bind more precious metals than the metal-free CCB support. Measurements were also performed after reuse tests (5×) of the catalysts and, in most cases, the metal content remained the same as before; thus, no significant metal leaching occurred (Table 1).

In one of our previous studies [32], non-carbonized cellulose beads were used for chlorate hydrogenation. The non-carbonized samples had low specific surface area (<1 m^2^/g) and low precious metal content (<0.6 wt%), and the Pd–Pt/CB catalyst achieved only a 72% chlorate conversion. Furthermore, metal leaching from the surface occurred. In contrast to the non-carbonized catalyst, the carbonized samples had a larger specific surface area (~400 m^2^/g), and the carrier–active metal interaction was stronger, as leaching did not occur.

**Figure 4:**
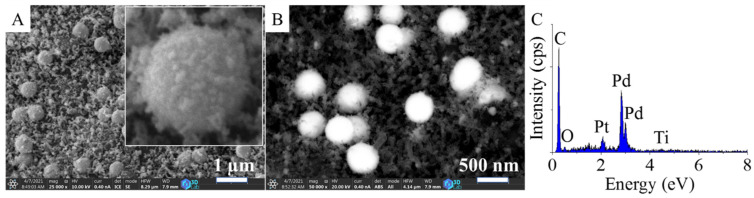
SEM images (**A**,**B**) and EDS spectrum (**C**) of the Pd–Pt/CCB catalyst.

The Pd–Pt/CCB catalyst was examined via SEM, and on the images, nanoparticle aggregates with a ~500 nm size were identified (Figure 4A,B). These spherical aggregates contained palladium and platinum particles together according to the elemental mapping (Supplementary Materials Figure S4). By the EDS analysis of the catalyst, the presence of palladium and platinum as catalytically active components and other elements. such as carbon, oxygen, and titanium, were verified (Figure 4C).

**Figure 5:**
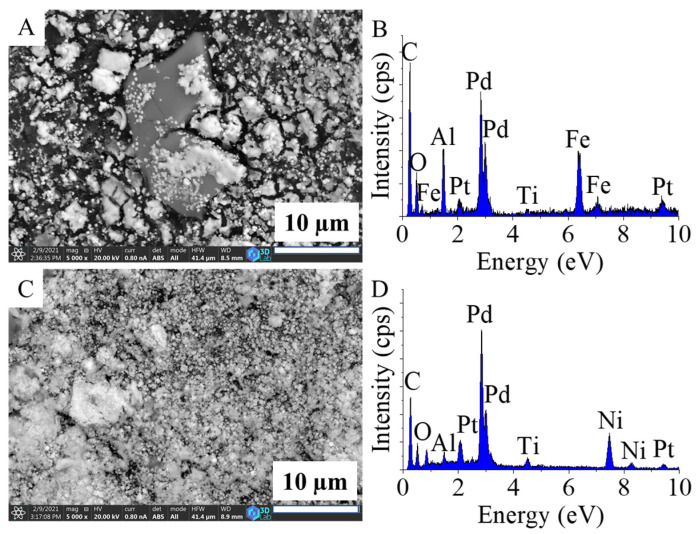
SEM images and EDS spectra of the Pd–Pt/Fe_3_O_4_–CCB (**A**,**B**) and Pd–Pt/Ni–CCB (**C**,**D**) catalysts.

The SEM analysis of the Pd–Pt/Fe_3_O_4_–CCB catalyst shows that aggregates formed on the surface in this case as well (Figure 5A). The EDS spectrum verified the presence of catalytically active metals, platinum, and palladium (Figure 5B). Additional peaks were associated with carbon, oxygen, iron, and Ti. Aluminum also appeared on the spectrum due to the sample holder of the SEM.

The Pd–Pt/Ni–CCB catalyst was also analyzed by SEM and, as in the case of the other two catalysts, metal particle aggregates were located on the surface (Figure 5C). The size of these aggregates were smaller compared to the ones on the surface of the Pd–Pt/Fe_3_O_4_–CCB catalyst. The presence of palladium, platinum, and nickel was confirmed by the EDS analysis (Figure 5D).

By increasing the magnification, it can be seen that the size of the spherical crystals of the precious metals were in the range of ~500–700 nm, and these larger aggregates were based on smaller nanoparticles of 10–20 nm (Supplementary Materials Figure S5). This special structure occurred for both Ni- and magnetite-promoted catalysts. The surface morphology of the Pd–Pt aggregates significantly differed from each other for the Pd–Pt/Fe_3_O_4_–CCB and Pd–Pt/Ni–CCB samples (Supplementary Materials Figure S5B,D). The precious metal spheres had a more closed, denser structure for the Pd–Pt/Fe_3_O_4_–CCB (Supplementary Materials Figure S5B) sample and a looser, more open structure in the case of Pd–Pt/Ni–CCB (Supplementary Materials Figure S4D). Furthermore, the size of the individual Pt and Pd nanoparticles of the aggregates were larger in the magnetite-containing sample compared to the nickel-promoted catalyst (Supplementary Materials Figure S5). The structural difference of the Pd–Pt crystallites cannot be explained by the type of precursor salts of the promoter metals, because in both cases nitrate salts were applied. However, the nickel was in elemental form, while the iron was in oxide form (as magnetite). As the magnetite and nickel particles on the surface of the CCB were in ideal places for crystal growth, they had an influence on the speed of nucleation and, thus, the structure of the crystallites, which build-up spherical aggregates in the catalysts.

**Figure 6:**
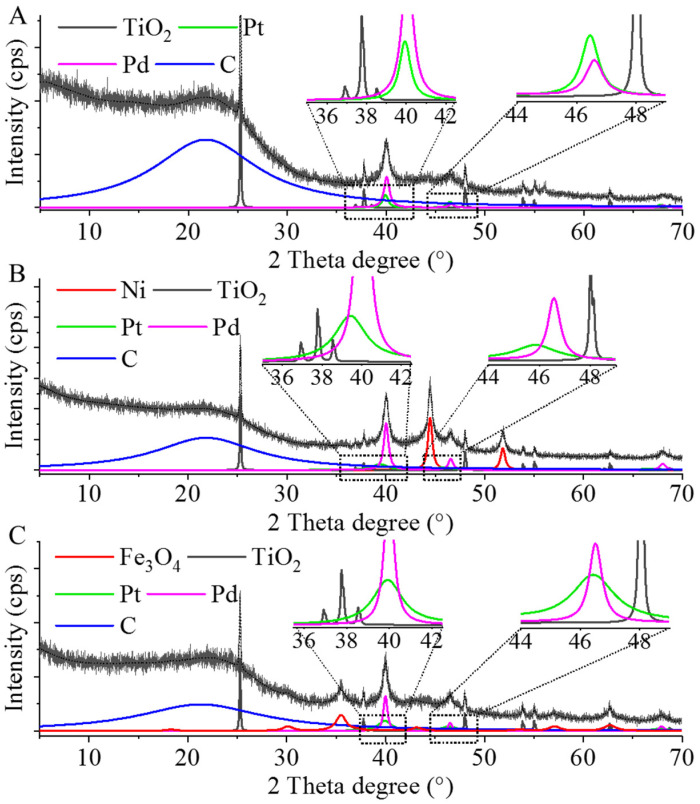
XRD patterns of the Pd–Pt/CCB (**A**), Pd–Pt/Ni–CCB (**B**), and Pd–Pt/Fe_3_O_4_–CCB (**C**) catalysts.

XRD measurement were also carried out to characterize the developed catalysts. On the diffractogram of the Pd–Pt/CCB, reflexions of elemental palladium were identified at 40.1° (111), 46.5° (200), and 68.1° (220), two theta degrees (JCPSD Card. No. 46-1043) (Figure 6A). The reflexions of Pt(111) and Pt(200) were also located at 39.4° and 45.9° 2ϴ degrees (JCPSD Card. No. 87-0640). On the diffractograms of the Pd–Pt/Ni–CCB and Pd–Pt/Fe_3_O_4_–CCB catalysts, the presence of palladium and platinum was verified (Figure 6B,C). Anatase was also identified in each catalyst, and the presence of nickel and magnetite was confirmed based on the corresponding XRD patterns (Figure 6B,C).

**Figure 7:**
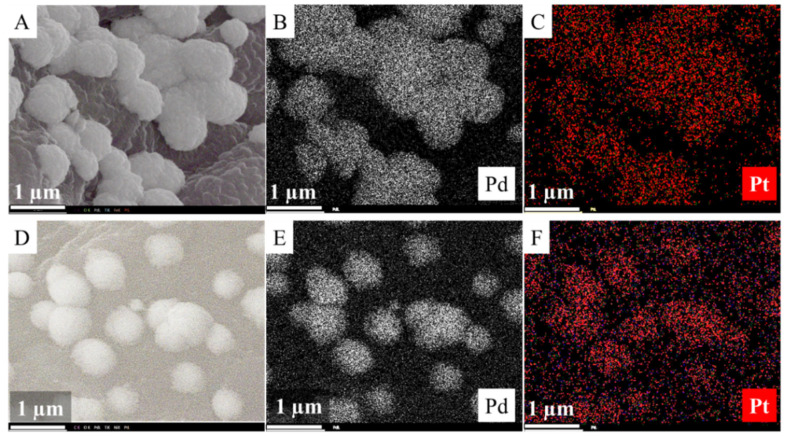
Elemental maps of the Pd–Pt/Fe_3_O_4_–CCB (**A**–**C**) and Pd–Pt/Ni–CCB (**D**–**F**) catalysts.

XRD measurements confirmed that palladium and platinum were present as individual nanoparticles in the catalysts, and they did not form an alloy. However, based on the results of the EDS mapping of the spherical aggregates (Supplementary Materials Figure S5), palladium and platinum nanoparticles were together in both catalysts (Figure 7).

FTIR measurements were performed after reuse tests of the Pd–Pt/CCB, Pd–Pt/Ni–CCB, and Pd–Pt/Fe_3_O_4_–CCB catalysts (Supplementary Materials Figure S6). Significant differences were not visible between the FTIR spectra of the catalysts before and after use in the case of the Pd–Pt/Ni–CCB and Pd–Pt/Fe_3_O_4_–CCB catalysts (Figure 2BC, Supplementary Materials Figure S6B,C). However, for the used Pd–Pt/CCB catalyst, the peaks representing the oxygen-containing functional groups (νC–O–C, νC–O, and βOH) significantly decreased (Supplementary Materials Figure S6A). Moreover, the –CH_2_ bands decreased compared to the carbonized cellulose beads.

The catalysts after reuse tests were also examined by XRD (Supplementary Materials Figures S7–S9). The XRD patterns of the fresh and used catalysts were compared. For the Pd–Pt/CCB and Pd–Pt/Fe_3_O_4_–CCB catalysts, there were no changes in the patterns before and after the reuse tests (Supplementary Materials Figures S7 and S8). However, in the case of the Pd–Pt/Ni–CCB catalyst, transformations occurred after the catalytic tests, and new phases formed including PdO, PtO_2_, and NiCO_3_ in small amounts (Supplementary Materials Figure S9). Small amounts of the noble metals were oxidized by the chlorate (as an oxidizing agent) and, thus, PdO and PtO_2_ formed. The presence of nickel carbonate can be explained by the formation of nickel oxide during the catalytic tests, which further transformed to NiCO_3_ by reacting with the CO_2_ content of the air during drying.

### 2.3. Catalytic Hydrogenation of Chlorate Ions in Aqueous Solution

The catalytic activity of the nickel- and magnetite-containing catalyst supports were measured (Supplementary Materials Figure S10). Both samples were active, but only a low conversion (Fe_3_O_4_–CCB 17.7 n/n% and Ni–CCB 42.4 n/n%) was achieved.

The three developed catalysts, Pd–Pt/CCB, Pd–Pt/Ni–CCB, and Pd–Pt/Fe_3_O_4_–CCB, were compared in hydrogenation of ClO_3_^–^ ions (Supplementary Materials Figure S11). The Pd–Pt/Ni–CCB sample was found to be the most catalytically active ($X{\%}_{KClO_{3}}$ > 99 n/n% after 45 min at 353 K) (Supplementary Materials Figure S11). Almost complete conversion was reached with the Pd–Pt/CCB catalyst, but more time was required (~3 h), while with the Pd–Pt/Fe_3_O_4_–CCB, only 84.6 n/n% was achieved after 3 h.

The enhanced catalytic activity of the nickel-promoted system can be explained by the structure of the Pd–Pt aggregates (Figure 4 and Supplementary Materials Figure S5). In case of the Ni-containing catalyst, the structure of the Pd–Pt crystals was opened, compared to the Pd–Pt/Fe_3_O_4_–CCB sample (Supplementary Materials Figure S5B,D). The structure of the aggregates in the Pd–Pt/Ni–CCB catalyst contained smaller, spherical precious metal nanoparticles that were easily accessible for the reactant molecules in the hydrogenation process. Thus, with the Ni-promoted sample, the H_2_ activation was facilitated, and a higher catalytic activity was achieved compared to the promoter-free or magnetite-containing sample.

**Figure 8:**
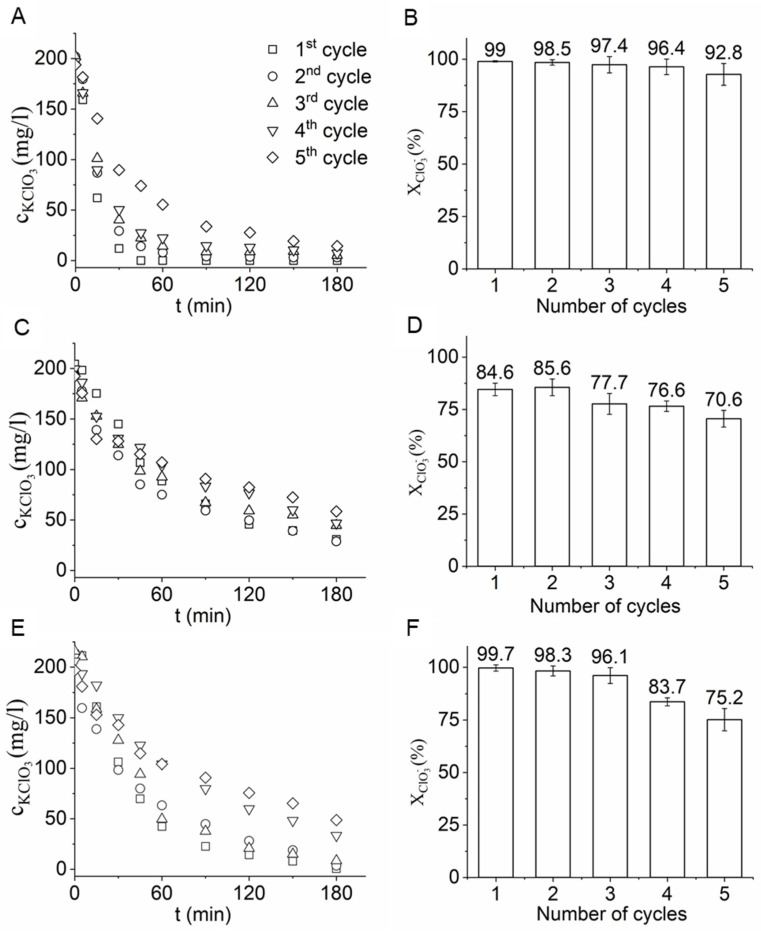
Chlorate conversion vs. time of hydrogenation using the Pd–Pt/Ni–CCB (**A**), Pd–Pt/Fe_3_O_4_–CCB (**C**), and Pd–Pt/CCB (**E**) catalysts. The chlorate conversion maximum after each reuse test cycle was determined for the Pd–Pt/Ni–CCB (**B**), Pd–Pt/Fe_3_O_4_–CCB (**D**), and Pd–Pt/CCB (**F**) catalysts.

The stability of the catalysts was also studied and compared by applying reuse tests (Figure 8). The nickel-promoted catalyst showed the highest chlorate conversion after five cycles; moreover, the difference in the chlorate conversion between the first and fifth cycles was only 6.2%. High conversion was achieved even though there was no regeneration (just a rinse and dry) between cycles (Figure 8A,B). In the case of the Pd–Pt/Fe_3_O_4_–CCB catalyst, deterioration was observed in the catalytic activity, as the chlorate conversion fell almost 14% during the 5th cycle (Figure 8C,D). An even more significant decrease in the chlorate conversion (24.5%) was observed for the Pd–Pt/CCB catalyst (Figure 8E,F).

The rate constants of the chlorate hydrogenation reactions when the developed Pd–Pt/Ni–CCB, Pd–Pt/Fe_3_O_4_, and Pd–Pt/CCB catalysts were applied were calculated using linear regression (Supplementary Materials Figure S12). The *k* values were also determined for the reuse tests (Table 2, Supplementary Materials Figure S13). The reaction rate during the reuse tests slightly decreased from 2.5 × 10^−4^ s^−1^ to 1.6 × 10^−4^ s^−1^ after five cycles in the case of the Pd–Pt/Fe_3_O_4_–CCB catalyst (Table 2). For the nickel-promoted catalyst, a more significant decrease was observed in the reaction rate, whereas the initial *k* value decreased from 1.6 × 10^−3^ s^−1^ to 3.7 × 10^−4^ s^−1^ after five cycles. The reaction rate also decreased when the Pd–Pt/CCB catalyst was applied. It must be noted that despite the decrease in the reaction rate, the Pd–Pt/Ni–CCB catalyst was able to remove >90% of the chlorate, even in the 5th cycle.

The catalysts were examined after the reuse tests, and chlorine was not detected in the samples (Supplementary Materials Figure S13). A simple wash and dry method was enough to reach a chlorine-free state of the catalysts, which were ready for reuse.

Thus, according to the results, the developed Pd–Pt/Ni–CCB, Pd–Pt/Fe_3_O_4_, and Pd–Pt/CCB catalysts are reusable, stable systems which can be applied efficiently in the hydrogenation of chlorate.

## 3. Materials and Methods

### 3.1. Materials

Mavicell (Hungarian Viscosa Corp., Nyergesújfalu, HU) was used to create carbonized cellulose beads (CCBs). Nickel(II) nitrate hexahydrate (Ni(NO_3_)_2_∙6H_2_O, Sigma–Aldrich Corp., Steinheim am Albuch, Germany) and iron(III) nitrate nonahydrate (Fe(NO_3_)_3_∙9H_2_O, Merck Chemicals GmbH, Darmstadt, Germany) were used as precursors of nickel and magnetite. Palladium(II) nitrate dihydrate (Pd(NO_3_)_2_∙2H_2_O, Alfa Aesar, Ltd., Heysham, UK) and platinum(IV) chloride (PtCl_4_, Sigma–Aldrich, Corp., Steinheim am Albuch, DE) were used as precursors of the Pd and Pt nanoparticles. Potassium chlorate (KClO_3_, Reanal Ltd., Budapest, Hungary), potassium iodide (KI, VWR Ltd., Radnor, PA, USA), and sulfuric acid (H_2_SO_4_, VWR Ltd., Radnor, PA, USA) were used during the chlorate hydrogenation experiments. 

### 3.2. Catalyst Preparation

Mavicell cellulose beads (5–5 g) were impregnated with an aqueous solution of nickel(II) nitrate and iron(III) nitrate. After drying (at 378.15 K), the impregnated beads were carbonized in a nitrogen atmosphere at 973.15 K. Non-impregnated cellulose beads were also carbonized for use as a reference. The impregnated and non-impregnated carbonized cellulose beads (CCBs) (1.14 g) were added to 50.00 mL aqueous solution of 0.10 g palladium(II) nitrate dihydrate and 0.011 g anhydrous platinum(IV) chloride. The precious metal ions were reduced to the elemental state using 50 mL hydrazine hydrate (4.00 wt%). Thus, three different catalysts, Pd–Pt/CCB, Pd–Pt/Ni–CCB, and Pd–Pt/Fe_3_O_4_–CCB, were prepared and dried at 378.15 K overnight.

### 3.3. Characterization Techniques

The particle size and morphology of the catalysts were studied using a Helios G4 PFIB CXe Plasma Focused Ion Beam Scanning Electron Microscope (PFIB-SEM) equipped with an EDAX Octane Elect EDS System with APEX Analysis Software using carbon tape for sample preparation. EDS maps were created with a 1024 × 800 resolution, and 1 frame was recorded with a 1000 μs collecting time. X-ray diffraction (XRD) analysis was also performed using a Bruker D8 Advance diffractometer (Cu-Kα source, 40 kV, and 40 mA with a Vantec 1 detector) to identify the nanoparticles. Thermogravimetric measurements were performed using a TG 209 F3 Tarsus with a 10 K/min heating rate starting from 308 K and increasing to 973.15 K in a nitrogen atmosphere. The functional groups of the carbonized cellulose beads were examined by Fourier transform infrared spectroscopy (FTIR) using Bruker Vertex 70 equipment. Measurements were carried out in transmission mode with KBr pellets (10 mg sample in 250 mg KBr), and the interval was 400–4000 cm^−1^, while the resolution was 4 cm^−1^ with a 16 min^−1^ scan rate. The specific surface area of the catalysts was determined by CO_2_ adsorption–desorption measurements using Micromeritics ASAP 2020 equipment and applying the Dubinin–Radushkevich isotherm model. The metal contents of the catalysts were measured using a Varian 720 ES inductively coupled optical emission spectrometer (ICP-OES). For the ICP-EOS measurements, the samples were ignited after preparation by placing them into an aqua regia.

### 3.4. Catalytic Hydrogenation of Chlorate

Hydrogenation of the ClO_3_^−^ ions was performed in an aqueous solution of potassium–chlorate (200 mg·dm^−3^). The solution was placed in a side-inlet gas washing bottle with fritted disc, and the temperature was set to 80 °C using a Julabo circulator. A gas supply was provided (40 sccm nitrogen and 100 sccm hydrogen), while the amount of catalyst was 200 mg for each measurement. The experiments were carried out for 3 h, and sampling was performed after 0, 5, 15, 30, 45, 60, 90, 120, 150, and 180 min. After the catalytic tests, the catalysts were washed with distilled water and dried at 105 °C overnight.

Chlorate concentration in the samples was determined using the UV-6300PC spectrophotometer at a 351 nm wavelength. The following redox reaction between iodide and chlorate ions were considered during the measurements:

$$KClO_{3}+6\;KI+6\;HCl\to \;3\;H_{2}O+3\;I_{2}+7\;KCl$$

Due to the iodine formation, the color intensity and, thus, the absorbance changed, which allowed for the determination of the chlorate concentration with appropriate calibration. Calibration was conducted using potassium–chlorate solutions with different concentrations (0, 50, 100, 150, and 200 mg∙dm^−3^). For each sample (1 mL), 100 mg potassium iodide and 1 mL hydrochloric acid were added, and then it was diluted to 50 mL with distilled water and measured with the spectrophotometer.

## 4. Conclusions

Catalysts were developed and carbonized cellulose beads were decorated with palladium–platinum nanoparticles using a chemical reduction method. Three different supports—pure CCBs and Ni- and magnetite-promoted CCBs—were synthesized and applied. They were decorated with Pd–Pt nanoparticles using a catalyst preparation process that was simple and efficient, and within which it was not necessary to apply time- and energy-consuming activations steps. The simultaneous decomposition of the precursors led to the formation of larger (500–600 nm) spherical aggregates that contained Pd and Pt together. Due to the fact of these bimetallic aggregates, the synergistic effect of the precious metals was more pronounced during the catalytic processes. By the application of nickel and magnetite as promoters, the structure of the aggregates changed, and in the case of the Pd–Pt/Ni–CCB catalyst, cavities formed on the surface. The Ni–CCB and Fe_3_O_4_–CCB supports were able to bind more precious metals than the metal-free CCB. Furthermore, the size of the individual Pt and Pd nanoparticles were smaller for the nickel-promoted catalyst. These were easily accessible for the reactant molecules in the hydrogenation process. Thus, with the Ni-promoted sample, the H_2_ activation was facilitated, and higher catalytic activity was achieved compared to the promoter-free or the magnetite-containing sample. By using the Pd–Pt/Ni–CCB catalyst, 99 n/n% chlorate conversion was reached after 45 min at 80 °C. For the Pd–Pt/Fe_3_O_4_–CCB catalyst, lower chlorate conversion (84.6 n/n %) was obtained after 3 h. During the reuse tests, the catalytic activity of the nickel-promoted bimetallic catalyst slightly decreased, and the ClO_3_^–^ conversion fell by only ~6% after five cycles. In contrast, the catalytic activity of the promoter-free (Pd–Pt/CCB) and magnetite-promoted (Pd–Pt/Fe_3_O_4_–CCB) systems decreased by 24.5% and ~14%, respectively. All in all, three catalysts were developed and successfully applied in chlorate hydrogenation. Although the Pd–Pt/Ni–CCB system is the most promising, the other two are also applicable for chlorate reduction.

## Figures and Tables

**Table 1:** Metal contents of the prepared Pd–Pt/CCB, Pd–Pt/Ni–CCB, and Pd–Pt/Fe_3_O_4_–CCB catalysts.

	Ni (wt%)	Fe_3_O_4_ (wt%)	Pd (wt%)	Pt (wt%)
Pd–Pt/Ni–CCB	8.97	-	2.03	0.29
Pd–Pt/Ni–CCB (5 × used)	8.85	-	2.06	0.29
Pd–Pt/Fe_3_O_4_–CCB	-	6.27	1.71	0.34
Pd–Pt/Fe_3_O_4_–CCB (5 × used)	-	6.48	1.72	0.35
Pd–Pt/CCB	-	-	0.96	0.19
Pd–Pt/CCB (5 × used)	-	-	0.95	0.18

**Table 2:** Reaction rate (*k*) constants after the reuse tests.

k (s^−1^)	Pd–Pt/Ni–CCB	Pd–Pt/Fe_3_O_4_–CCB	Pd–Pt/CCB
1st cycle	1.6 × 10^−^^3^ ± 1.2 × 10^−4^	2.5 × 10^−4^ ± 1.3 × 10^−5^	4.2 × 10^−4^ ± 2.3 × 10^−5^
2nd cycle	9.5 × 10^−4^ ± 5.5 × 10^−5^	2.7 × 10^−4^ ± 1.8 × 10^−5^	3.4 × 10^−4^ ± 1.9 × 10^−5^
3rd cycle	7.7 × 10^−4^ ± 4.3 × 10^−5^	2.2 × 10^−4^ ± 1.7 × 10^−5^	3.1 × 10^−4^ ± 2.8 × 10^−5^
4th cycle	6.3 × 10^−4^ ± 5.5 × 10^−5^	1.8 × 10^−4^ ± 1.7 × 10^−5^	1.7 × 10^−4^ ± 5.3 × 10^−6^
5th cycle	3.7 × 10^−4^ ± 2.2 × 10^−5^	1.6 × 10^−4^ ± 1.8 × 10^−5^	1.3 × 10^−4^ ± 1.0 × 10^−5^

## Data Availability

Not applicable.

## Supplementary Materials

They are available online at www.mdpi.com/article/10.3390/ijms222111846/s1.
