# Supplementary material for: Development of Nickel- and Magnetite-Promoted Carbonized Cellulose Bead-Supported Bimetallic Pd–Pt Catalysts for Hydrogenation of Chlorate Ions in Aqueous Solution
Source: Int J Mol Sci. 2021 Oct 31;22(21):11846. doi: 10.3390/ijms222111846 (PMC8584269; doi:10.3390/ijms222111846)
Supplement: Supplementary file 1 [file ijms-22-11846-s001.zip › ijms-1428992-supplementary.pdf]

Development of nickel and magnetite promoted carbonized cellulose beads supported bimetallic Pd-Pt catalysts for hydrogenation of chlorate ions in aqueous solution

Emőke Sikora[1,2,a], Dániel Koncz-Horváth[2,b], Gábor Muránszky[1,2,c], Ferenc Kristály[3,d], Béla Fiser[1,2,4,e], Béla Viskolcz[1,f], László Vanyorek[1,g*]

[1]Institute of Chemistry, University of Miskolc, 3515 Miskolc-Egyetemváros, Hungary
[2]Higher Education Industry Cooperation Centre, University of Miskolc, 3515 Miskolc-Egyetemváros, Hungary
[3]Institute of Mineralogy and Geology, University of Miskolc, 3515 Miskolc-Egyetemváros, Hungary
[4]Ferenc Rákóczi II. Transcarpathian Hungarian College of Higher Education, 90200 Beregszász, Transcarpathia, Ukraine

[*] Corresponding author
[a]kemsik@uni-miskolc.hu, [b]femkhd@uni-miskolc.hu, [c]kemmug@uni-miskolc.hu, [d]askkf@uni-miskolc.hu, [e]kemfiser@uni-miskolc.hu, [f]bela.viskolcz@uni-miskolc.hu, [g]kemvanyi@uni-miskolc.hu

SUPPLEMENTARY INFORMATION

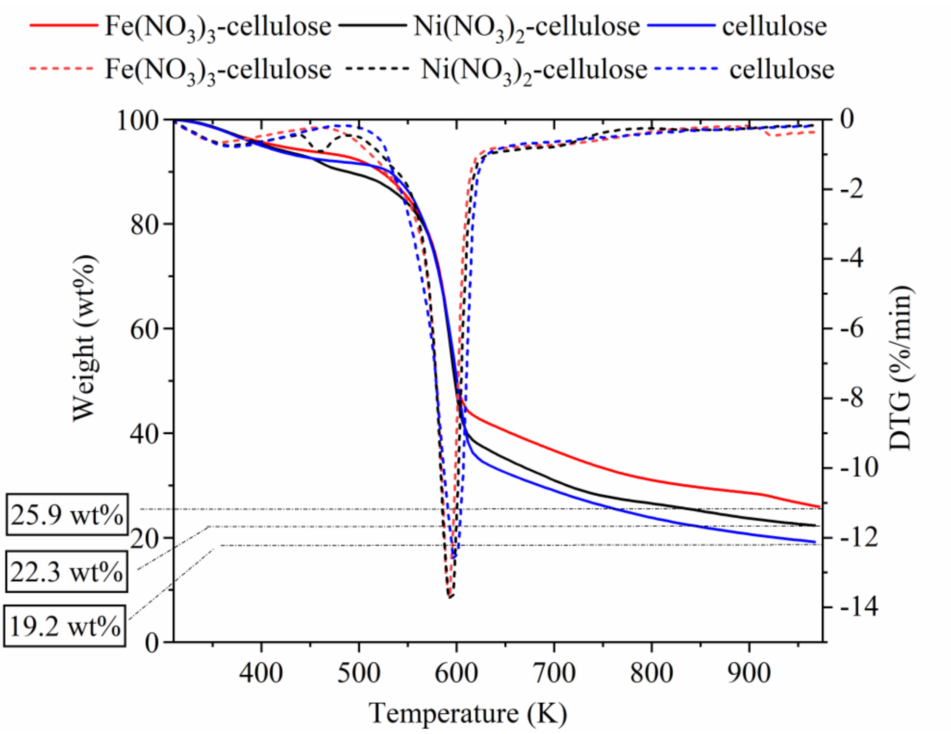

Figure S1. TG-DTG curves of the impregnated (with iron(III) nitrate or nickel(II) nitrate) cellulose beads (CB) and the non-impregnated sample.

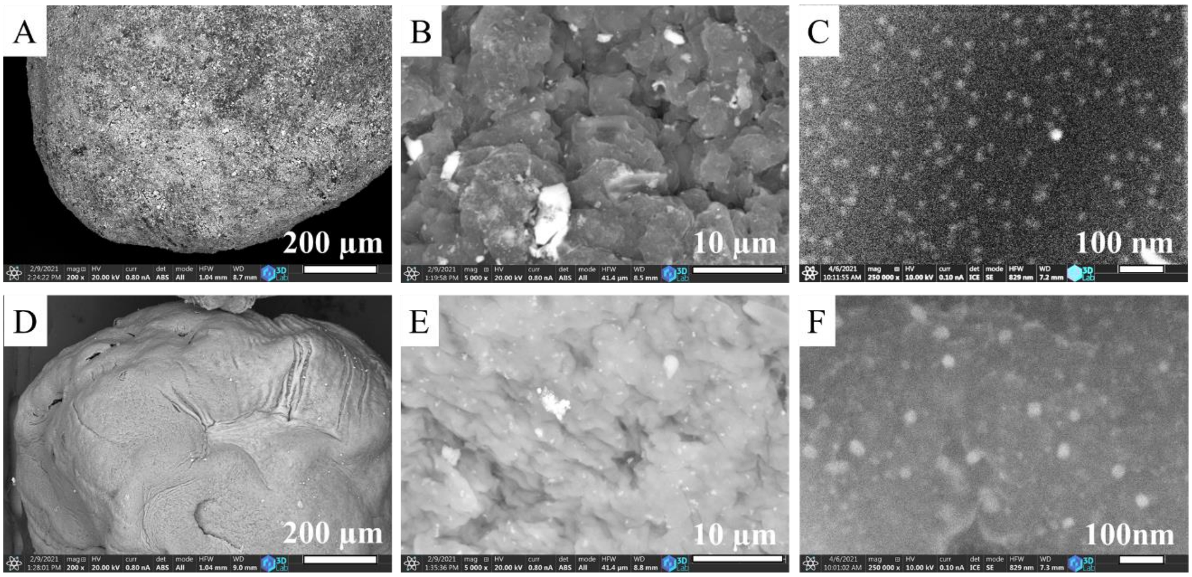

Figure S2. SEM images of the surface morphology of the Fe_3O_4-CCB (A-C), and Ni-CCB (D-F) catalyst supports.

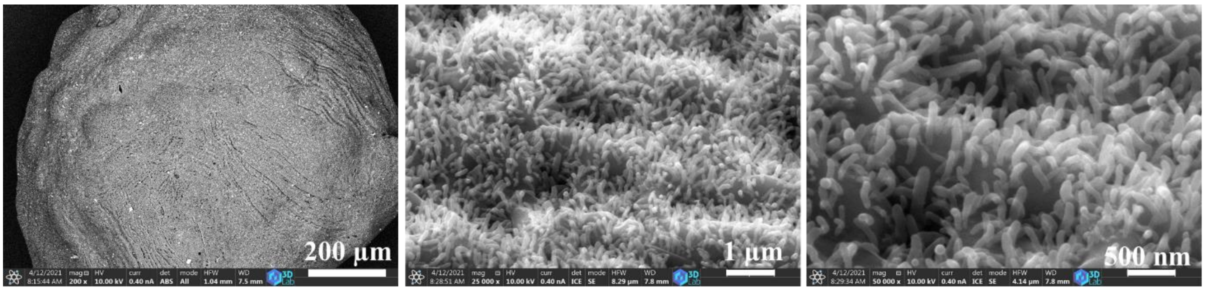

Figure S3. SEM images of the surface morphology of the non-impregnated carbonized cellulose bead (CCB) catalyst support.

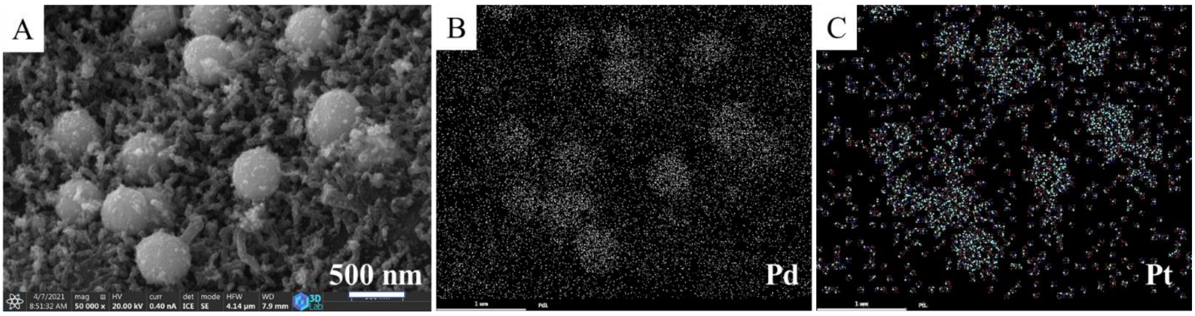

Figure S4. Elemental maps of the aggregated particles on the surface of the Pd-Pt/CCB catalyst with the localization of palladium (B) and platinum (C).

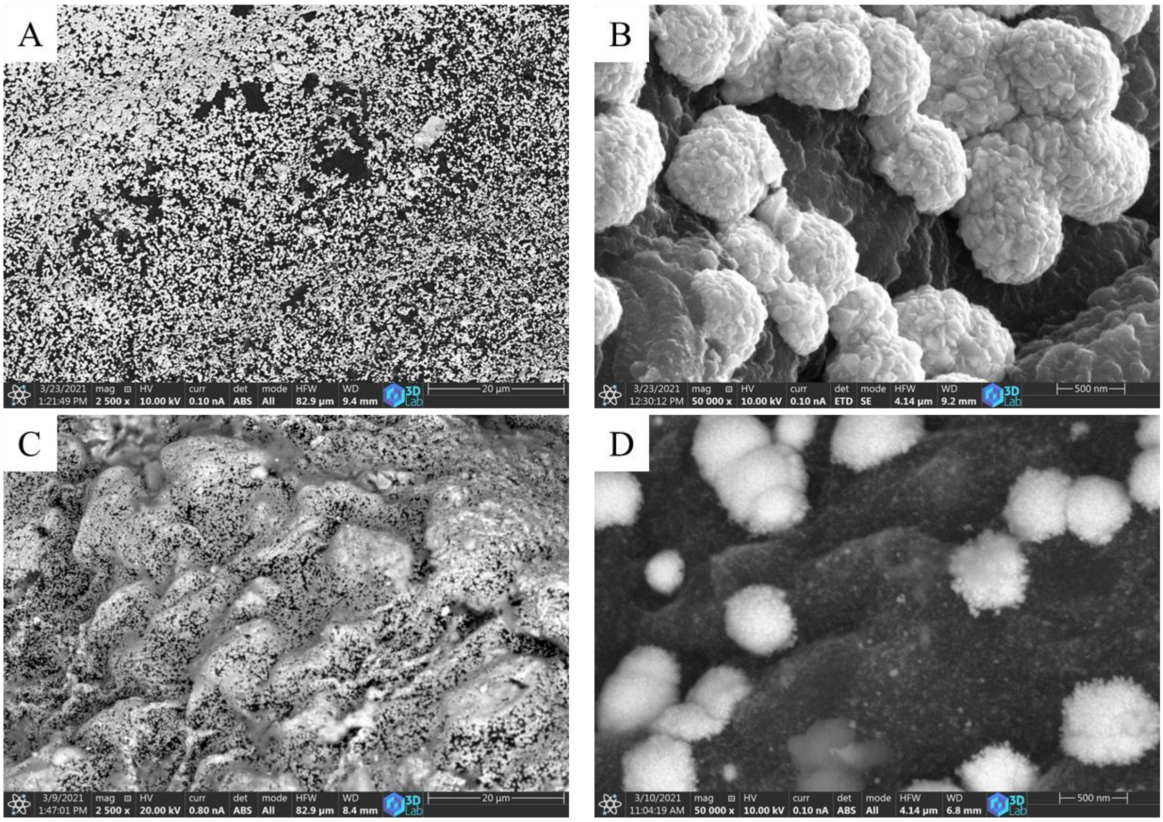

Figure S5. SEM images of the palladium and platinum nanoparticle based aggregated crystallites on the surface of the Fe_3O_4-CCB (A, B) and Ni-CCB (C, D) catalyst supports shown at different magnifications.

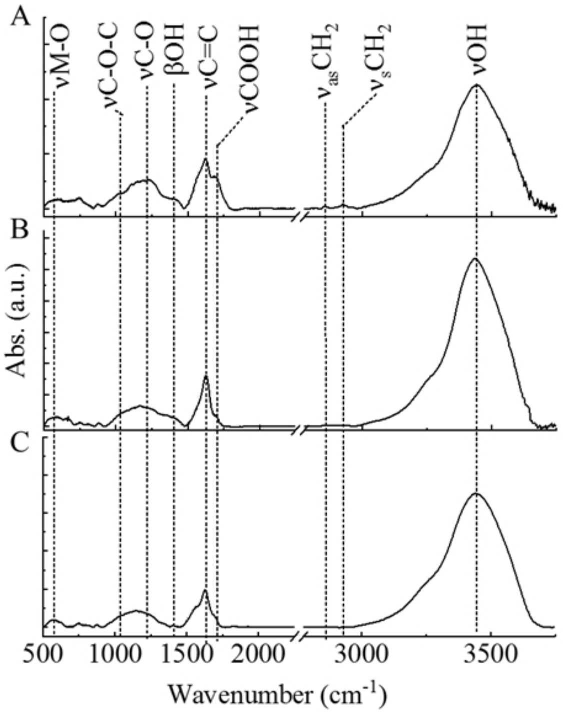

Figure S6. FTIR spectra of the 5 times used Pd-Pt/CCB (A), the Pd-Pt/Ni-CCB (B), Pd-Pt/Fe_3O_4-CCB (C) catalysts.

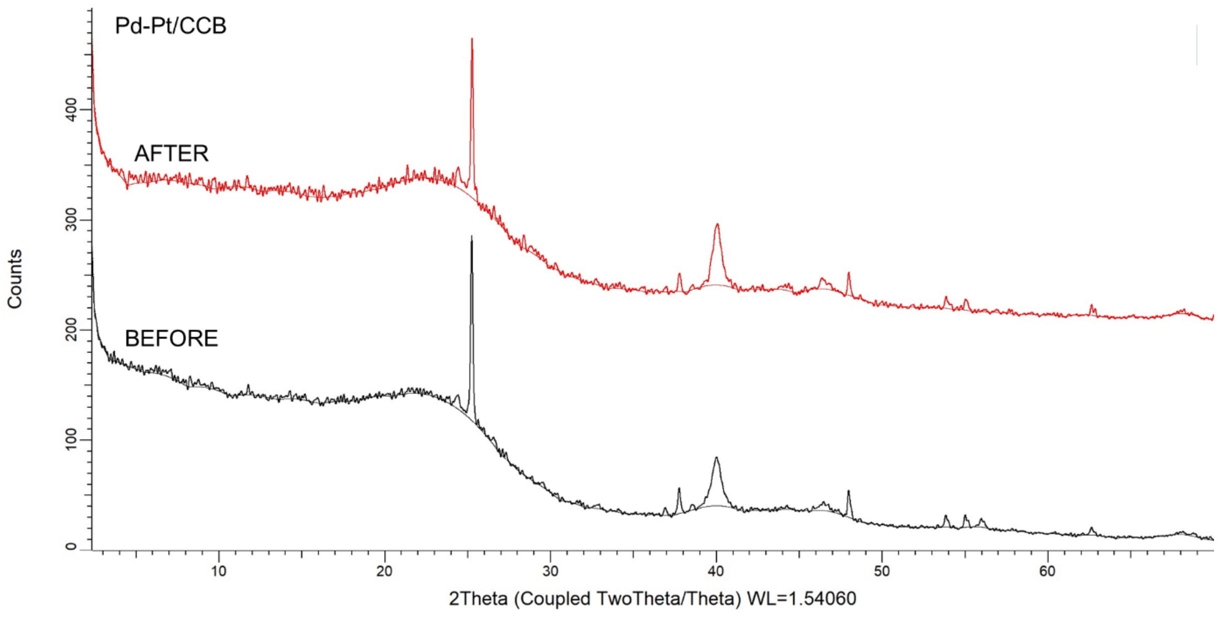

Figure S7. XRD patterns of the fresh (before) and the 5 times used (after) Pd-Pt/CCB catalyst.

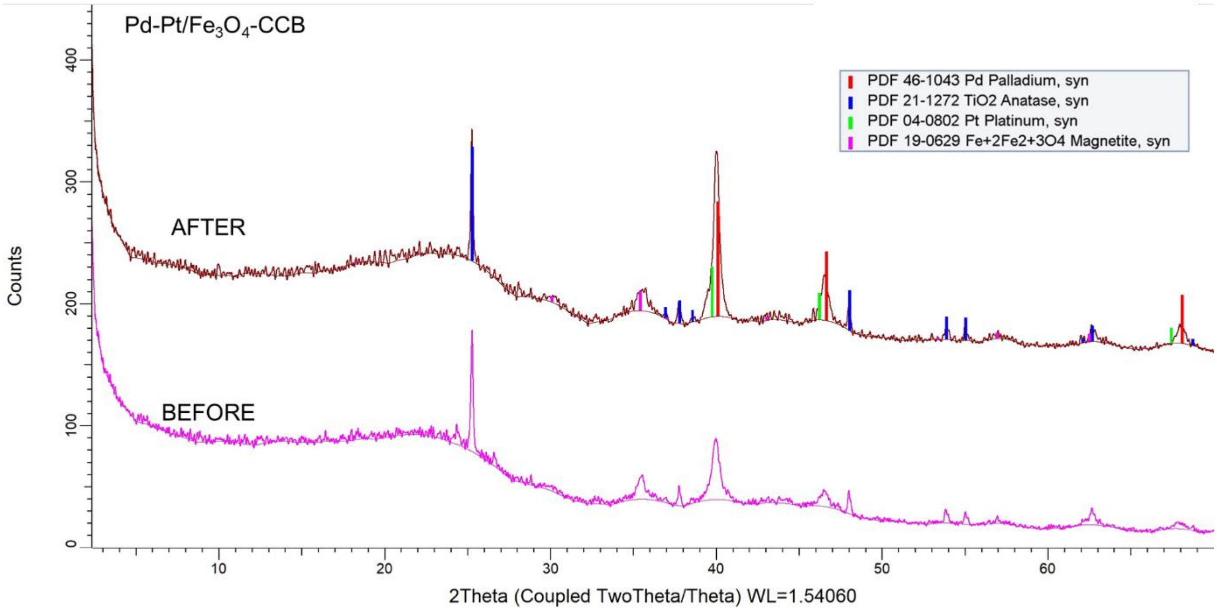

Figure S8. XRD patterns of the fresh (before) and the 5 times used (after) Pd-Pt/Fe_3O_4-CCB catalyst.

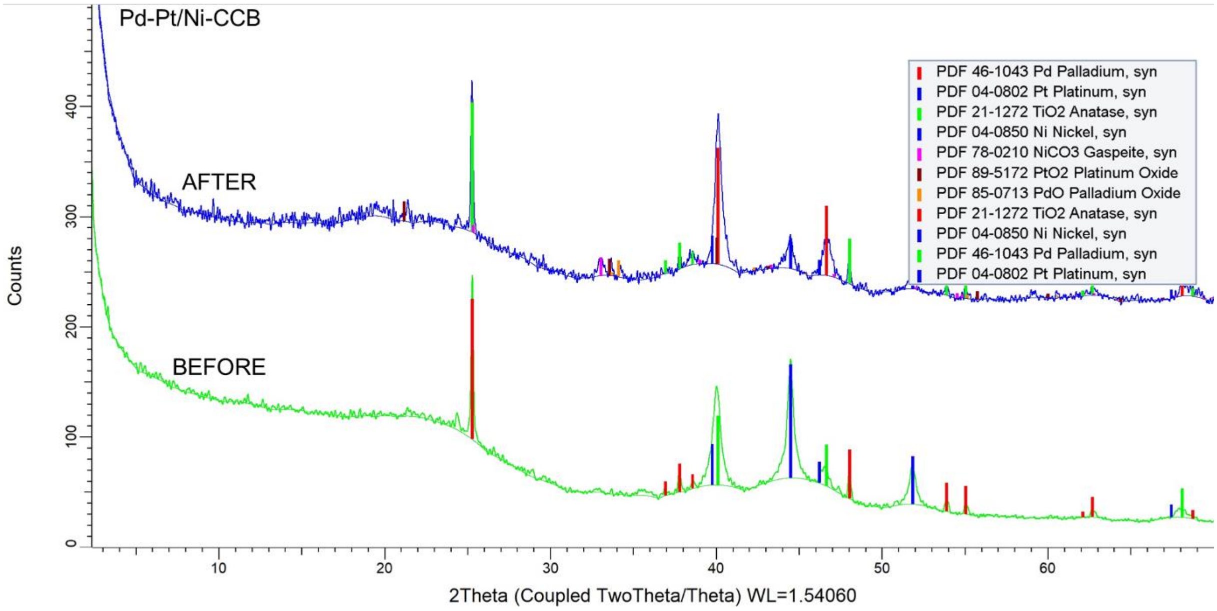

Figure S9. XRD patterns of the fresh (before) and the 5 times used (after) Pd-Pt/Ni-CCB catalyst.

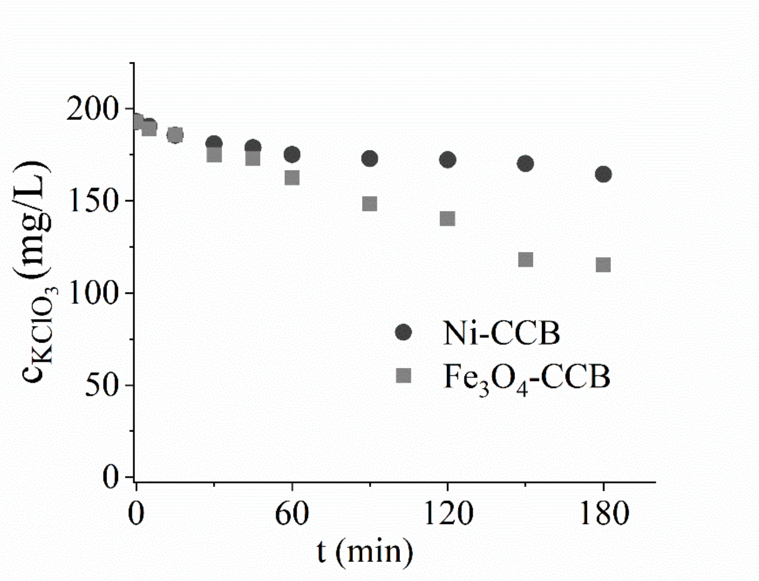

Figure S10. Chlorate conversion *vs.* time of hydrogenation. Catalytic activity of the Ni-CCB and Fe_3O_4-CCB supports.

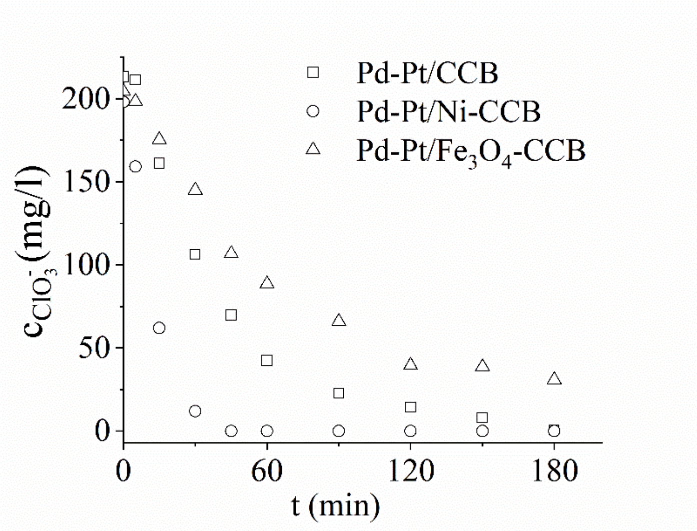

Figure S11. Chlorate conversion *vs.* time of hydrogenation by using the developed Pd-Pt/CCB, Pd-Pt/Ni-CCB, and Pd-Pt/Fe_3O_4-CCB catalysts.

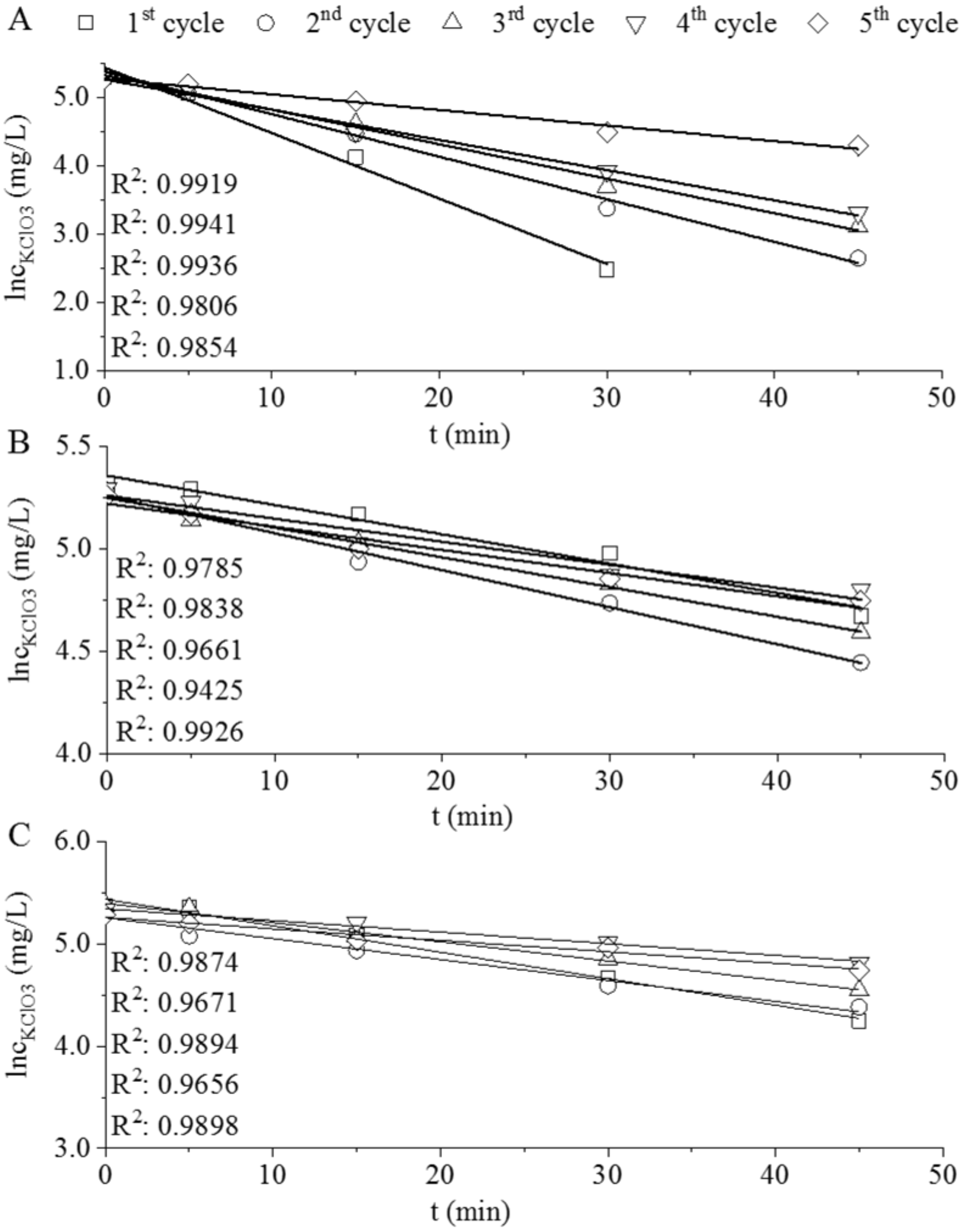

Figure S12. ln*c* *vs*. time (min) after the reuse tests by using the Pd-Pt/Ni-CCB (A), Pd-Pt/Fe_3O_4-CCB (B), and Pd-Pt/CCB (D) catalysts.

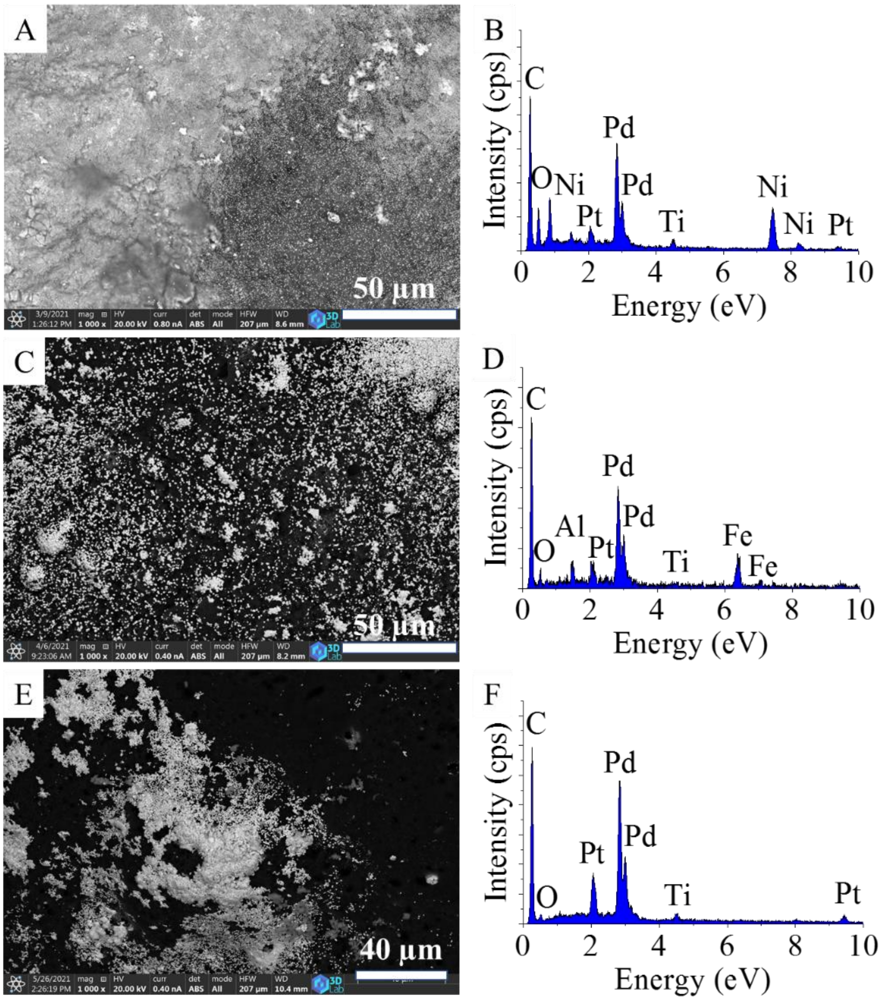

Figure S13. SEM images and EDS spectra of the Pd-Pt/Fe_3O_4-CCB (A, B), Pd-Pt/Ni-CCB (C, D), and Pd-Pt/CCB (E, F) catalyst after five reuse cycles.
